# Clinical Importance of Focal Adhesion Kinase (FAK)-Src and Paxillin Expression in Renal Cell Carcinoma

**DOI:** 10.7759/cureus.62706

**Published:** 2024-06-19

**Authors:** Panagiotis Mitsos, Ioannis Anastasiou, Constantinos Constantinides, Dimitrios Deligiannis, Ioannis Katafigiotis, Anna Papakonstantinou, Vasiliki Tzotzola, Dionysios Mitropoulos, Stamatios Theocharis

**Affiliations:** 1 Department of Urology, Agia Sofia Children's Hospital, Athens, GRC; 2 First Department of Urology, Laiko Hospital, Medical School, National and Kapodistrian University of Athens, Athens, GRC; 3 Department of Urology, Naval Hospital of Athens, Athens, GRC; 4 Department of Laparoscopy and Endourology, Central Urology, Lefkos Stavros, The Athens Clinic, Athens, GRC; 5 Department of Pediatric Hematology-Oncology, Agia Sofia Children's Hospital, Athens, GRC; 6 First Department of Pathology, Medical School, National and Kapodistrian University of Athens, Athens, GRC

**Keywords:** tyrosine kinase, src, paxillin, focal adhesion kinase, renal cell carcinoma, kidney tumor

## Abstract

Background and objective: The complex focal adhesion kinase (FAK)/Src and paxillin seem to play a key role in the pathogenesis and progression of cancer. The aim of this study is to evaluate the expression of these proteins in renal cell carcinomas (RCCs), considering the immunoreactive score (IRS), the positivity and the intensity, and to find any association with patients' clinical characteristics, histologic type and other pathological features that imply a possible pathophysiological or prognostic role of FAK/Src and paxillin in RCC.

Methods: Patients with RCC who had undergone partial or radical nephrectomy from January 2009 to September 2010 were eligible for this retrospective cross-sectional study. The immunohistochemical expression of FAK, Src and paxillin proteins in formalin-fixed paraffin-embedded tumour tissue was analysed in association with various clinicopathological features.

Results: Out of ninety patients, 58 had clear cell renal carcinoma, 15 had papillary, 11 had chromophobe and six had unclassified RCC. FAK, Src and paxillin were expressed in 55.6%, 32.2% and 18.9% of all cases, respectively. In univariate analysis, FAK positivity and IRS were more likely in patients with papillary and chromophobe histologic type versus clear cell RCC (p<0.005), Src positivity and IRS presented more frequently in stage T3 versus T1 (p<0.005) and paxillin positivity was more likely in patients with stage T3 versus T2 (p=0.021) and grades 3-4 versus grade 2 (p=0.013). Paxillin-IRS was not associated with any clinicopathological features. The same associations were also reproduced in the multifactorial analysis for the FAK and Src positivity and IRS, while it was found that paxillin positivity and IRS were associated with the female gender (p=0.052, p=0.024), and were higher in grades 3-4 versus grade 2 (p=0.022, p=0.020).

Conclusions: Our study suggests that RCC shows immunohistochemical expression of FAK, Src and paxillin proteins, and this expression varies in relation to the histologic type, the stage and the stage/grade/gender, respectively. These findings imply a possible involvement of the FAK/Src signalling pathway in the pathogenesis and progression of cancer in RCC, providing future perspectives for targeted therapies with inhibitors.

## Introduction

Renal cell cancer (RCC) is one of the most common urological cancers and represents up to 5% of all cancers [1]. Despite the early diagnosis, up to 30% of the patients with RCC have already distant metastasis at the time of diagnosis, and an additional 20% will develop metastases after radical surgical treatment, making renal cell cancer the most lethal urological cancer [2]. Established risk factors for RCC include lifestyle factors such as smoking, obesity and hypertension [3-5].

Based on genetic and cytogenetic analyses, the three main histological types are papillary, chromophobe cell and clear cell renal carcinoma, accounting for 70% of all cases. At the same time, the 2022 WHO classification has additionally introduced newly molecular-defined entities [6].

Localised RCC tumours are usually well-circumcised, and partial or radical nephrectomy is the appropriate treatment, either with open surgery or laparoscopically/robot-assisted technique. When indicated, adjuvant therapy is administered, achieving a five-year disease-specific survival rate of 55-91%. In contrast, metastatic RCC has a poor prognosis despite multimodal therapeutic with a five-year disease-specific survival ranging from 0 to 32% [7]. In the last decades, newer agents such as immunotherapy and checkpoint inhibitors, have been widely used to improve the outcome [7-9]. In parallel, efforts focus on understanding the pathogenesis of RCC and the use of tumour biological characteristics for prognosis, risk stratification and therapy guidance.

One of the pathways of interest is the focal adhesion kinase (FAK)/Src-paxillin signalling. Focal adhesion kinase (FAK) and Src are both non-receptor tyrosine kinases that interact as a dual kinase complex, contributing to cancer progression and genesis of metastases. FAK is a focal adhesion-associated protein and it is regulated by b-integrin and growth factor receptors resulting in its autophosphorylation at the Y397 domain. Activated FAK binds to Src family kinases in the SH2 domain, leading to phosphorylation of FAK and further activation of the kinase activity. The complex FAK/Src also phosphorylates later scaffolds of the signalling pathway, such as paxillin, enhancing the motility, migration, survival, proliferation and overall, the spread of malignant cells [10,11].

For this study, we hypothesised that FAK, Src and paxillin proteins contribute to the genesis and progression of RCC, and therefore they are immunohistochemically expressed in the tumour tissue and positively correlate with more advanced staging and other clinicopathological features.

## Materials and methods

Our study is a retrospective observational cross-sectional study. We included 90 patients who were diagnosed with kidney tumours and treated surgically either with partial nephrectomy or with radical nephrectomy in the General Hospital of Athens, Laiko, in the first University Urological Clinic, Medical School of Athens. The histological samples were examined from the First Department of Pathology, Medical School, National and Kapodistrian University of Athens, from January 2009 to September 2010, which confirmed the diagnosis of RCC tumour. Further immunohistochemical analysis with tissue blocks in paraffin was made in the above neoplasms using the following kits and antibodies: (1) BOND Refine Polymer Detection KIT LEICA Biosystems Newcastle Ltd, (2) Anti-p-paxillin (Tyr, sc-101774) Santa Cruz Biotechnology, USA, (3) Anti FAK (H-1, sc-1688), Santa Cruz Biotechnology, USA, and (4) Anti c-Src (H-12, sc-5266) Santa Cruz Biotechnology, USA.

The immunoreactive score (IRS) was used to assess the expression of the examined proteins (positive cell score and intensity of staining score) [12].

Data were expressed as mean ± SD or median and interquartile range (IQR) for quantitative variables and as frequencies (n) and percentages (%) for categorical variables. Unifactorial analyses were made by using the student t-test and Chi-square test or Fisher exact test to analyse the relation between the categorical outcome variables (FAK positivity (negative vs. positive), Src positivity (negative vs. positive), paxillin positivity (negative vs. positive), FAK immunohistochemical score (0-1 vs. 2-8), Src immunohistochemical score (0-1 vs. 2-8), and paxillin immunohistochemical score (0-1 vs. 2-8)) and the quantitative and qualitative demographic and clinical variables, respectively. Variables in the univariate analysis were further assessed in a multifactorial binary logistic regression model with an enter method to identify independent demographic and clinical predictors of the outcome variables. All assumptions of regression models (homoscedasticity, linearity, normality and independence of error terms, as well as multicollinearity of independent variables) were examined. All tests are two-sided, and statistical significance was set at p<0.05. All analyses were carried out using the statistical package SPSS version 21 (IBM Corporation, Somers, NY, USA).

## Results

In our study, 63 (70%) patients were male and 27 (30%) were female, with a mean age of 59.96 years. Concerning the stage of the disease, in 74 patients (82.2%) the tumour was localised at the kidney and in 16 patients (17.8%) the tumour extended to adjacent tissues such as the major veins or other perinephric tissues. Fifty-eight patients had clear cell tumours (64.4%), 15 papillary tumours (16.7%), 11 chromophobe tumours (12.2%) and six unclassified tumours. Regarding the grade of these tumours, nine of them were G1 (10%), 75 were G2 or G3 (83.3%) and six of them were G4 (6.7%). Only 4 (4.4%) patients had lymph node infiltration. Table 1 shows the demographic and clinical characteristics of our group. The expression of FAK, Src and paxillin was positive in 55.6%, 32.2% and 18.9% of the cases, respectively.

**Table 1:** Demographic and clinical characteristics. FAK: focal adhesion kinase, IRS: immunoreactive score.

	n (%)
Histologic type	
Clear cell	58 (64.4)
Papillary	15 (16.7)
Chromophobe	11 (12.2)
Unclassified	6 (6.37)
Grade	
Grade 1	9 (10)
Grade 2	45 (50)
Grade 3	30 (33.3)
Grade 4	6 (6.7)
Gender	
Male	63 (70)
Female	27 (30)
Stage Τ	
T1	63 (70)
T2	11 (12.2)
T3	16 (17.8)
Stage N	
N0	86 (95.6)
N1	2 (2.2)
N2	2 (2.2)
FAK negative	40 (44.4)
Positive	50 (55.6)
FAK intensity 1	44 (88)
2	6 (12)
3	0 (0)
Src negative	61 (67.8)
Positive	29 (32.2)
Src intensity 1	24 (82.8)
2	5 (17.2)
3	0 (0)
Paxillin negative	73 (81.1)
Positive	17 (18.9)
Paxillin intensity 1	10 (58.8)
2	7 (41.2)
3	0 (0)
Age mean ± SD (range)	59.96 ± 12.96 (25-85)
FAK (%), mean ± SD (range)	28.54 ± 18.01 (7-65)
Src (%), mean ± SD (range)	23.45 ± 10.70 (10-50)
Paxillin (%), mean ± SD (range)	25.88 ± 13.26 (10-60)
FAK IRS 0-1	51 (56.7)
2-3	33 (36.7)
4-8	6 (6.7)
Src IRS 0-1	65 (72.2)
2-3	20 (22.2)
4-8	5 (5.6)
Paxillin IRS 0-1	74 (82.2)
2-3	9 (10.0)
4-8	7 (7.8)

Tables 2-4 show the unifactorial analysis of demographic and clinical characteristics in relation to the positivity and IRS score for FAK, Src and paxillin.

**Table 2:** Unifactorial analysis of demographic and clinical characteristics in relation to FAK positivity and IRS score. All categorical variables were presented as frequencies (%). FAK: focal adhesion kinase, IRS: immunoreactive score.

Variables		FAK positivity		p-value	FAK IRS score		p-value
		Negative (n=40)	Positive (n=50)		No (n=51)	Weak-moderate (n=39)	
Age; mean ± SD		60.03 ± 11.96	59.90 ± 13.83	0.964	60.18 ± 12.03	59.67 ± 14.24	0.854
Histologic type	Clear cell	33 (56.9)	25 (43.1)	<0.005	40 (69.0)	18 (31.0)	<0.005
	Papillary	2 (13.3)	13 (86.7)		4 (26.7)	11 (73.3)	
	Chromophobe	2 (18.2)	9 (81.8)		4 (36.4)	7 (63.6)	
Grade	1	6 (66.7)	3 (33.3)	0.234	6 (66.7)	3 (33.3)	0.074
	2	21 (46.7)	24 (53.3)		30 (66.7)	15 (33.3)	
	3 and 4	13 (36.1)	23 (63.9)		15 (41.7)	21 (58.3)	
Gender	Male	29 (46)	34 (54)	0.817	13 (48.1)	14 (51.9)	0.355
	Female	11 (40.7)	16 (59.3)		38 (60.3)	25 (39.7)	
Stage Τ	1	28 (44.4)	35 (55.6)	0.996	36 (57.1)	27 (42.9)	0.774
	2	5 (45.5)	6 (54.5)		7 (63.6)	4 (36.4)	
	3	7 (43.8)	9 (56.3)		8 (50)	8 (50)	

**Table 3:** Unifactorial analysis of demographic and clinical characteristics in relation to Src positivity and IRS score. All categorical variables were presented as frequencies (%). IRS: immunoreactive score.

Variables		Src positivity		p-value	Src IRS score		p-value
		Negative (n=61)	Positive (n=29)		No (n=65)	Weak-moderate (n=25)	
Age; mean ± SD		60.49 ± 12.45	58.83 ± 14.14	0.572	60.75 ± 12.46	57.88 ± 14.24	0.349
Histologic type	Clear cell	42 (72.4)	16 (27.6)	0.351	45 (77.6)	13 (22.4)	0.306
	Papillary	8 (53.3)	7 (46.7)		9 (60.0)	6 (40)	
	Chromophobe	7 (63.6)	4 (36.4)		7 (63.6)	4 (36.4)	
Grade	1	7 (77.8)	2 (22.2)	0.795	7 (77.8)	2 (22.2)	0.920
	2	30 (66.7)	15 (33.3)		32 (71.1)	13 (28.9)	
	3 and 4	24 (66.7)	12 (33.3)		26 (72.2)	10 (27.8)	
Gender	Male	42 (66.7)	21 (33.3)	0.809	21 (77.8)	6 (22.2)	0.608
	Female	19 (70.4)	8 (29.6)		44 (69.8)	19 (30.2)	
Stage Τ	1	49 (77.8)	14 (22.2)	<0.005	52 (82.5)	11 (17.5)	<0.005
	2	7 (63.6)	4 (36.4)		7 (63.6)	4 (36.4)	
	3	5 (31.3)	11 (68.8)		6 (37.5)	10 (62.5)	

**Table 4:** Unifactorial analysis of demographic and clinical characteristics in relation to paxillin positivity and IRS score All categorical variables were presented as frequencies (%). IRS: immunoreactive score.

Variables		Paxillin positivity		p-value	Paxillin IRS score		p-value
		Negative (n=73)	Positive (n=17)		No (n=74)	Weak-moderate (n=16)	
Age; mean ± SD		60.16 ± 11.98	59.06 ± 16.96	0.753	60.15 ± 11.89	59.06 ± 17.52	0.763
Histologic type	Clear cell	50 (86.2)	8 (13.8)	0.348	50 (86.2)	8 (13.8)	0.486
	Papillary	11 (73.3)	4 (26.7)		11 (73.3)	4 (26.7)	
	Chromophobe	8 (72.7)	3 (27.3)		9 (81.8)	2 (18.2)	
Grade	1	7 (77.8)	2 (22.2)	0.045	7 (77.8)	2 (22.2)	0.081
	2	41 (91.1)	4 (8.9)		41 (91.1)	4 (8.9)	
	3 and 4	25 (69.4)	11 (30.6)		26 (72.2)	10 (27.8)	
Gender	Male	53 (84.1)	10 (15.9)	0.378	20 (74.1)	7 (25.9)	0.231
	Female	20 (74.1)	7 (25.9)		54 (85.7)	9 (14.3)	
Stage Τ	1	52 (82.5)	11 (17.5)	0.044	52 (82.5)	11 (17.5)	0.113
	2	11 (100)	0 (0)		11 (100.0)	0 (0)	
	3	10 (62.5)	6 (37.5)		11 (68.8)	5 (31.3)	

There was a statistically significant association between histologic type and FAK positivity (p<0.005). Patients with papillary (p<0.005) and chromophobe (p<0.005) histologic type presented a higher likelihood of positive expression of FAK compared with those with clear cell type, as shown in Table 1. There were statistically significant associations between stage T and Src positivity (p<0.005). As shown in Table 2, patients with stage T3 (p<0.005) were more likely to present positive expression of Src compared with those with stage T1. Moreover, there were statistically significant associations between grade (p=0.045), stage T (p=0.044) and paxillin positivity. Specifically, positive expression of paxillin was more frequent in patients with stage T3 (p=0.021) compared with those with stage T2, and in patients with grades 3-4 (p=0.013) than those with grade 2 (Table 4).

Our study also showed that there was a statistically significant association between histologic type and FAK IRS (p<0.005). In other words, weak or moderate expression of FAK IRS was higher in patients with papillary (p<0.005) and chromophobe (p=0.039) histologic types than in those with clear cell type. Statistically significant associations were also found between stage T and Src IRS (p<0.005) since the patients with stage T3 (p<0.005) presented a higher likelihood of weak or moderate expression of Src IRS compared with those with stage T1. No statistically significant associations were shown between the demographic and clinical characteristics of the sample and paxillin IRS.

Multifactorial analysis was also performed, as shown in Tables 5-7. Multiple logistic regression models with the enter method (all variables in the unifactorial analysis were included in the model) were employed to examine the effect of demographic and clinical variables to qualitative outcomes (FAK positivity, Src positivity, paxillin positivity, FAK IRS, Src IRS and paxillin IRS). All models satisfied all assumptions of logistic regression analysis. The model showed a statistical trend Χ2(9)=15.97 p=0.067, accounting for 21.8% (Nagelkerke R2) of the variance of FAK positivity. Patients with papillary (OR (95%CI): 9.1 (1.8-46.2); p=0.008) and chromophobe (OR (95%CI): 5.3 (0.2-2.9); p=0.053) histologic type presented a higher likelihood of positive expression of FAK compared with those with clear cell type (Table 5).

**Table 5:** Multifactorial analysis of demographic and clinical characteristics in relation to FAK positivity and IRS score. OR: odds ratio, CI: confidence interval, FAK: focal adhesion kinase, IRS: immunoreactive score.

	Reference category	FAK positivity				FAK IRS score			
		OR	95%CI		p-value	OR	95%CI		p-value
Age		1.01	0.97	1.05	0.705	0.99	0.95	1.03	0.700
Gender (female)	Male	1.43	0.52	3.95	0.488	2.40	0.84	6.88	0.102
Grade					0.515				0.064
2	1	1.35	0.28	6.56	0.710	0.43	0.08	2.26	0.317
3 and 4		2.23	0.43	11.72	0.342	1.52	0.28	8.13	0.625
Stage Τ					0.930				0.852
2	1	0.83	0.20	3.52	0.801	0.65	0.14	2.93	0.572
3		0.81	0.23	2.90	0.744	0.92	0.25	3.41	0.900
Histologic type					0.020				0.013
Papillary	Clear cell	9.04	1.77	46.27	0.008	9.44	2.26	39.43	<0.005
Chromophobe		5.31	0.98	28.75	0.053	3.41	0.75	15.46	0.112
Unclassified		1.10	0.19	6.41	0.912	2.37	0.39	14.54	0.352

The model was statistically significant Χ2(9)=17.40 p=0.043, accounting for 24.6% (Nagelkerke R2) of the variance of Src positivity. Patients with stage T3 (OR (95%CI): 13.6 (3.2-57.9); p<0.005) presented a higher likelihood of positive expression of Src (4.4%) compared with those with stage T1 (Table 6).

**Table 6:** Multifactorial analysis of demographic and clinical characteristics in relation to Src positivity and IRS score. OR: odds ratio, CI: confidence interval, IRS: immunoreactive score.

	Reference category	Src positivity				Src IRS score			
		OR	95%CI		p-value	OR	95%CI		p-value
Age		1.00	0.95	1.03	0.498	0.98	0.93	1.02	0.277
Gender (female)	Male	0.75	0.23	2.43	0.633	0.45	0.12	1.74	0.248
Grade					0.497				0.283
2	1	0.94	0.16	5.71	0.948	0.60	0.09	3.89	0.595
3 and 4		0.47	0.07	3.38	0.455	0.24	0.03	1.95	0.180
Stage Τ					<0.005				<0.005
2	1	2.23	0.52	9.52	0.281	3.23	00.70	14.85	0.133
3		13.60	3.19	57.94	<0.005	19.48	4.02	94.40	<0.005
Histologic type					0.465				0.393
Papillary	Clear cell	2.94	0.77	11.30	0.115	3.09	0.73	13.15	0.126
Chromophobe		1.55	0.31	7.54	0.593	2.51	0.46	13.74	0.290
Unclassified		1.14	0.13	10.28	0.908	2.04	0.20	20.76	0.547

The model was statistically significant Χ2(9)=20.15, p=0.010, accounting for 32.3% (Nagelkerke R2) of the variance of paxillin positivity. Females (OR (95%CI): 4.20 (0.99-17.9); p=0.052) presented a higher likelihood of positive expression of paxillin compared with males. Patients with grade 2 (OR (95%CI): 0.18 (0.04-0.78); p=0.022) presented lower percent positive expression of paxillin compared with those with grades 3-4 (Table 7).

**Table 7:** Multifactorial analysis of demographic and clinical characteristics in relation to paxillin positivity and IRS score. OR: odds ratio, CI: confidence interval, IRS: immunoreactive score.

	Reference category	Paxillin positivity				Paxillin IRS score			
		OR	95%CI		p-value	OR	95%CI		p-value
Age		0.97	0.933	1.02	0.239	0.97	0.92	1.02	0.174
Gender (female)	Male	4.20	0.990	17.90	0.052	5.70	1.26	25.80	0.024
Grade					0.040				0.037
2	1	2.06	0.25	17.16	0.504	1.99	0.23	17.33	0.533
3 and 4		0.18	0.04	0.78	0.022	0.17	0.04	0.75	0.020
Stage Τ					0.576				0.869
2	1				0.999	0.00	0.00		0.998
3		2.15	0.52	8.98	0.294	1.49	0.34	6.43	0.596
Histologic type					0.241				0.195
Papillary	Clear cell	4.96	0.86	28.69	0.074	5.35	0.92	31.12	0.062
Chromophobe		2.33	0.34	16.00	0.389	1.03	0.13	8.41	0.980
Unclassified		4.98	0.57	43.33	0.146	5.72	0.65	49.93	0.115

Regarding the IRS scores, the multifactorial analysis showed statistical significance for FAK and Src. The model was statistically significant Χ2(9)=19.79, p=0.019, accounting for 26.5% (Nagelkerke R2) of the variance of FAK IRS patients with papillary (OR (95%CI): 9.5 (2.3-39.4); p<0.005) histologic type presented a higher likelihood of weak or moderate expression of FAK IRS compared with those with clear cell type (Table 5). The model was statistically significant Χ2(9)=20.78, p=0.014, accounting for 29.7% (Nagelkerke R2) of the variance of Src IRS patients with stage T3 (OR (95%CI): 19.5 (4.0-94.4); p<0.005) presented a higher likelihood of weak or moderate expression of Src IRS compared with those with stage T1 (Table 6). In contrary with the unifactorial analysis, the model was statistically significant Χ2(9)=18.93 p=0.026, accounting for 31.2% (Nagelkerke R2) of the variance of paxillin IRS. Females (OR (95%CI): 5.7 (1.3-25.8); p=0.024) presented a higher likelihood of weak or moderate expression of paxillin IRS compared with males. Patients with grade 2 (OR (95%CI): 0.17 (0.04-0.75); p=0.020) were less likely to present weak or moderate expression of paxillin IRS compared with those with grades 3-4 (Table 7).

## Discussion

RCC is the most common renal cancer in adults, and although early stages have a favourable prognosis with current therapeutic options, the outcome in advanced and metastatic stages remains dismal. During the last decade, many efforts have been made to interfere in the signalling pathways involved in the pathogenesis of RCC. Early studies aimed to enhance immune-mediated mechanisms by administering cytokines such as IL-2 and INF-γ [13]. In the early 1990s, a better understanding of RCC biology led to targeting angiogenesis, using antiangiogenic agents, including anti-vascular endothelial growth factor receptor (VEGFR) tyrosine kinases (sunitinib, sorafenib) and anti-vascular endothelial growth factor (VEGF) monoclonal antibody, bevacizumab. mTOR inhibitors (temsirolimus, everolimus) have also been added in the armamentarium against RCC targeting cancer progress and angiogenesis, while immune checkpoint inhibitors against PD-1 and CTLA4, such as nivolumab, ipilimumab are recently tested in various combinations to achieve durable responses and improve the disease-free survival. Nevertheless, a recent meta-analysis has shown that only pembrolizumab improves disease-free survival in RCC following nephrectomy when compared to the placebo group, and none of the studied drugs improves overall or recurrence-free survival [14]. The response rate remains low, ranging from 5-39% and the median progression-free survival is 11 months, making a deeper understanding of the RCC pathogenesis an imperative necessity [15].

The FAK/Src dual kinase complex and its signalling cascade seem to play a significant role in tumorgenesis and chemotherapy resistance in many cancer types, such as head and neck, colon, breast, prostate, liver, thyroid, gliomas, pancreatic and others [16]. In several studies [16-18], a negative association between kinases’ expression and prognosis was observed. For example, concomitant FAK, Src and paxillin tumour positivity and myelocytomatosis oncogene (MYCN) amplification had statistically significant increased mortality in children with neuroblastoma [19]. Immunohistochemical profiling of the tumour with FAK/Src and paxillin expression could have prognostic as well as therapeutic potential.

Our study showed frequent FAK, Src and paxillin positivity in RCC tissue, suggesting it is noteworthy to further investigate the role of FAK/Src signalling pathway and its cascade, in the cancer progression and migration of renal cell carcinoma, especially in papillary and chromophobe histologic types. The higher likelihood of Src expression in stage T3 and paxillin expression in stage T3 and grades 3-4 may imply their role in progression to more advanced stages, or it may be associated with a more aggressive tumour profile. Further studies are needed to study the survival of patients with RCC in correlation with FAK, Src and paxillin expression to clarify if a prognostic value exists in RCC as well. The correlation of female gender with paxillin expression, as found in the multifactorial analysis, should be validated in future studies with a larger number of patients. 

Preclinical studies have already highlighted that the inhibition of the FAK/Src signalling pathway could result in therapeutic benefits in various types of cancer. More specifically, by impairing the FAK/Src and paxillin interactions, the phosphorylation of FAK and FAK substrates decreases, resulting in blockage of cell adhesion, migration and invasion [20, 21]. Regarding RCC, several preclinical studies on cell lines show similar findings. Antcin-H, a fungus-derived product, showed a dose-dependent decrease in phosphorylated FAK and paxillin levels [22]. In another study, simultaneous targeting of Src kinase and receptor tyrosine kinase with saracatinib and sunitinib resulted in up to 80% blockade of RCC cell migration, synergistic inhibition of cell growth and reduction of acquired drug resistance [23]. Additional studies at the preclinical level support that targeting the FAK scaffold is a promising approach for developing novel therapies for RCC [24-26].

Currently, many early-phase clinical trials have been developed to study the effect of the FAK/Src pathway by pharmaceutical agents in several hematological malignancies and solid tumours in adults [27]. Future results remain to show the clinical value of FAK/Src inhibition also in patients with metastatic RCC, whose prognosis remains unfavourable. 

Regarding the limitations of our study, the number of patients enrolled was small (n=90) and single-centered. Moreover, we did not include a control group of healthy patients, to evaluate the expression of the examined proteins also in these patients. Additionally, the follow-up of the sample was not recorded to investigate associations with survival. Further studies need to verify the expression of FAK, Src and paxillin proteins in RCC and define in larger samples its prognostic value and the therapeutic value. 

## Conclusions

Immunohistochemical analysis in our study showed that FAK, Src and paxillin proteins are expressed in a portion of the renal cell carcinoma cases. Clinicopathological features such as histologic type, stage, grade and gender could further define a subgroup of patients with a higher likelihood of expression of these proteins.

These findings suggest a possible involvement of the FAK/Src signalling pathway and its substrate, paxillin, in the pathogenesis and progression of cancer in RCC. Future preclinical and clinical studies should investigate the clinical efficacy of FAK/Src inhibition with targeted therapies, aiming to improve the survival of patients with RCC.
